# Strategic Design and Mechanistic Understanding of Vacancy‐Filling Heusler Thermoelectric Semiconductors

**DOI:** 10.1002/advs.202407578

**Published:** 2024-09-03

**Authors:** Weimin Hu, Song Ye, Qizhu Li, Binru Zhao, Masato Hagihala, Zirui Dong, Yubo Zhang, Jiye Zhang, Shuki Torri, Jie Ma, Binghui Ge, Jun Luo

**Affiliations:** ^1^ School of Materials Science and Engineering Shanghai University Shanghai 200444 China; ^2^ Institutes of Physical Science and Information Technology Anhui University 111 Jiulong Road Hefei 230601 China; ^3^ Key Laboratory of Artificial Structures and Quantum Control School of Physics and Astronomy Shanghai Jiao Tong University Shanghai 200240 China; ^4^ Institute of Materials Structure Science High Energy Accelerator Research Organization (KEK) Tokai Ibaraki 3191106 Japan; ^5^ Minjiang Collaborative Center for Theoretical Physics College of Physics and Electronic Information Engineering Minjiang University Fuzhou 350108 China; ^6^ Interdisciplinary Materials Research Center School of Materials Science and Engineering Tongji University Shanghai 201804 China

**Keywords:** half‐Heusler, semiconductor, Slater‐Pauling rule, thermoelectric materials, vacancy‐filling

## Abstract

Doping narrow‐gap semiconductors is a well‐established approach for designing efficient thermoelectric materials. Semiconducting half‐Heusler (HH) and full‐Heusler (FH) compounds have garnered significant interest within the thermoelectric field, yet the number of exceptional candidates remains relatively small. It is recently shown that the *vacancy‐filling approach* is a viable strategy for expanding the Heusler family. Here, a range of near‐semiconducting Heuslers, TiFe*_x_* Cu*_y_* Sb, creating a composition continuum that adheres to the Slater‐Pauling electron counting rule are theoretically designed and experimentally synthesized. The stochastic and incomplete occupation of vacancy sites within these materials imparts continuously changing electrical conductivities, ranging from a good semiconductor with low carrier concentration in the endpoint TiFe_0.67_Cu_0.33_Sb to a heavily doped p‐type semiconductor with a stoichiometry of TiFe_1.00_Cu_0.20_Sb. The optimal thermoelectric performance is experimentally observed in the intermediate compound TiFe_0.80_Cu_0.28_Sb, achieving a peak figure of merit of 0.87 at 923 K. These findings demonstrate that vacancy‐filling Heusler compounds offer substantial opportunities for developing advanced thermoelectric materials.

## Graphical abstract

By filling a defined amount of Cu atoms to the tetrahedral interstices, the multiphase TiFeSb alloy is stabilized into TiFe*_x_* Cu*_y_* Sb semiconductors with the HH‐like structure. Owing to the enhanced Seebeck coefficient and reduced thermal conductivity, TiFe_0.80_Cu_0.28_Sb achieves a thermoelectric figure of merit of 0.87 at 923 K.

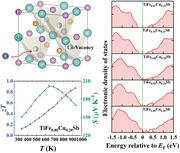

## 1 Introduction

Thermoelectric materials, which convert waste heat into electricity, contend with practical limitations in achieving an optimal figure‐of‐merit ($zT\;=\frac{S^{2}\sigma }{\kappa_{e}+\kappa_{L}}\;T$; where *S* is the Seebeck coefficient, *σ* is the electrical conductivity, *κ*_e_ is the electronic thermal conductivity, *κ*_L_ is the lattice thermal conductivity, and *T* is temperature) due to the inherently conflicted material attributes. A critical balance is required in the electronic properties, especially with carrier density (*n*): a smaller *n* is beneficial for an effective *S*, yet a higher *n* is crucial for maximizing *σ*.^[^ ^1^ ^]^ Sophisticated band engineering techniques have been developed,^[^ ^2^ ^]^ and good thermoelectrics are typically narrow‐gap semiconductors that are heavily doped with charge carriers.^[^ ^3^ ^]^ In addition, several successful advances have been demonstrated in reducing *κ*_L_.^[^ ^4^ ^]^ While substitution with isoelectronic elements introduces mass fluctuations that effectively decrease *κ*_L_, further introducing rattling atoms can reduce *κ*_L_ to near glassy limits. However, existing materials often demonstrate the principles effectively but cannot optimize all attributes simultaneously. The ongoing quest for better thermoelectric materials continues.

Heusler alloys^[^ ^5^ ^]^ have emerged as multifunctional materials with applications spanning various fields, including thermoelectrics. These alloys are distinguished by a face‐centered cubic structure, which facilitates flexible functional manipulation.^[^ ^6^ ^]^ HHs, represented by the formula XYZ where X and Y are transition metals and Z is a main‐group element, exhibit a significant electronegativity difference between X and Z. This difference results in a NaCl‐type structure: X at the Wyckoff position 4a (0,0,0), Z at 4b (1/2,1/2,1/2), and Y at 4c (1/4,1/4,1/4). This arrangement effectively fills half of the tetrahedral voids within the rock‐salt framework.^[^ ^7^ ^]^ Progressing to FHs, where all tetrahedral interstices are occupied, the stoichiometry changes to XY_2_Z. HH and FH have 18 and 24 valence electrons for the semiconducting systems, respectively, and each atom has six on average. Known as the Slater‐Pauling electron counting rule,^[^ ^8^, ^9^, ^10^ ^]^ it has been instrumental in discovering new semiconductors. Well‐studied thermoelectric candidates include XNiSn,^[^ ^11^, ^12^, ^13^, ^14^, ^15^ ^]^ and XCoSb (X = Ti, Zr, Hf),^[^ ^16^, ^17^, ^18^ ^]^ ZrCoBi,^[^ ^19^, ^20^ ^]^ XFeSb,^[^ ^21^, ^22^, ^23^, ^24^ ^]^ and XCoSn (X = V, Nb, Ta).^[^ ^25^, ^26^ ^]^

To broaden the range of Heusler semiconductors, two strategies appear to explore Heusler semiconductors that adhere to the 18‐electron rule. One approach involves modifying 19‐electron HH to optimize the carrier concentration by creating cation vacancies,^[^ ^27^, ^28^ ^]^ resulting in *off‐stoichiometric HH‐like semiconductors*. For instance, Nb_0.8_CoSb^[^ ^29^, ^30^ ^]^ and Ti_0.75_NiSb^[^ ^31^ ^]^ demonstrate impressive thermoelectric efficiencies. Another strategy involves blending 17‐ and 19‐electron HH systems to achieve an averaged 18‐electron configuration, known as *double‐HH semiconductors*. An example is TiFe_0.5_Ni_0.5_Sb, first theoretically proposed by Jeff Snyder's team^[^ ^32^ ^]^ and later successfully synthesized.^[^ ^33^ ^]^

Recent studies from a few groups,^[^ ^34^, ^35^ ^]^ including our own,^[^ ^36^, ^37^, ^38^, ^39^, ^40^, ^41^ ^]^ have demonstrated the potential of a *vacancy‐filling approach* to enhance the diversity of Heusler compounds. This method entails the partial occupations of 4d sites with element Y, forming off‐stoichiometric compounds XY_1+_ *_δ_* Z (0 < *δ* < 1). For example, for the Ti‐Fe‐Sb systems, where the 17‐electron HH TiFeSb is experimentally absent, Tavassoli et al. successfully synthesized polycrystal TiFe_1.33_Sb using the vacancy‐filling approach.^[^ ^34^ ^]^ Our research on TiFe_1.33_Sb reveals that its thermoelectric properties are accompanied by exotic non‐Fermi‐liquid behavior.^[^ ^37^ ^]^ Similarly, Huang et al. developed XCo_1.5_Sn (X = Ti, Zr, Hf) and evaluated the thermoelectric properties.^[^ ^35^ ^]^ Despite the unique properties of TiFe_1.33_Sb and XCo_1.5_Sn, they are classified as metals since they do not conform to the Slater‐Pauling electron counting rule. Toward thermoelectric applications, we have designed several semiconducting systems.^[^ ^39^, ^40^, ^41^ ^]^ For example, in the XRu_1+_ *_δ_* Sb systems (where X = Ti, Zr, Hf), electrical conductivities can be tuned from p‐type to n‐type, showcasing promising thermoelectric properties.^[^ ^39^, ^40^, ^41^ ^]^ The partial and typically random occupation of 4d sites in these *vacancy‐filling* systems has significant implications for their thermoelectric performance, distinguishing them from the *off‐stoichiometric HH‐like* and *double‐HH* systems.

Using the vacancy‐filling strategy, we here present an alternative pathway to stabilize Ti‐Fe‐Sb systems. The principle involves introducing additional electrons into the 17‐electron matrix of TiFeSb by doping it with copper. Unlike the scenario in Nb_0.8_CoSb, the Cu atom at a 4d site in our materials not only contributes an electron (resulting in a *d* ^10^ shell) but also plays a pivotal role in stabilizing the Heusler structure. Our theoretical guidelines predict various quaternary TiFe*_x_* Cu*_y_* Sb semiconductors, spanning from HH TiFe_0.67_Cu_0.33_Sb with *x* + *y* = 1 and 18 electrons to FH TiFeCuSb with *x* + *y* = 2 and 28 electrons. All systems are semiconductors in theory, but disordered vacancy occupations can diminish the bandgap and develop some metallic behavior. Many systems, which exhibit favorable formation energies in theory, are then successfully synthesized in our experiments. Agreeing with the prediction, we experimentally observe that the carrier density (*n*) increases proportionately to the vacancy occupation (*x* + *y*). Notably, the best thermoelectric performance is achieved in an intermediate composition, TiFe_0.80_Cu_0.28_Sb, which reached a peak thermoelectric figure‐of‐merit of 0.87 at 923 K. These results underscore the efficacy of the vacancy‐filling approach as a versatile method for developing advanced thermoelectric materials.

## 2 Results and Discussion

### 2.1 Theoretical Design of TiFe*_x_* Cu*_y_* Sb Semiconductors

**Figure 1:**
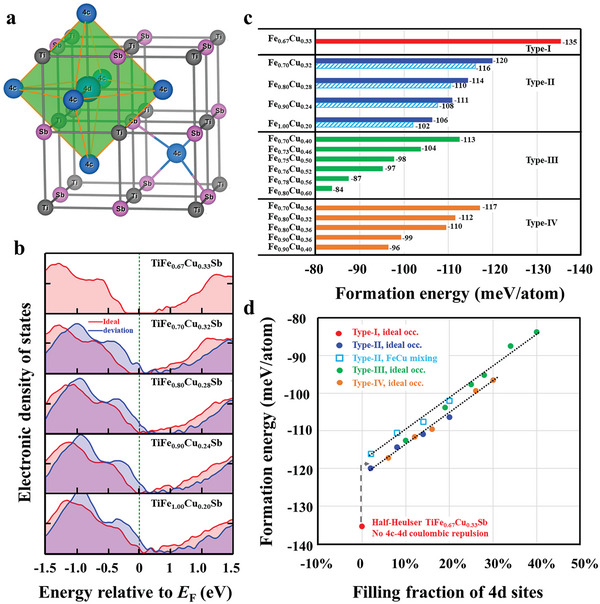
Structural, electronic, and energetic properties of TiFe*_x_* Cu*_y_* Sb. a) Structural model of vacancy filling technique. b) Electronic density‐of‐states of Type‐I and Type‐II systems. The red lines correspond to structures with ideal Fe and Cu occupations, whereas the blue lines represent minor deviations from the occupation rule. c) The formation energy of example systems. For Type‐II systems, the dashed bars represent structures with Fe‐Cu occupation irregularities. d) Formation energy plotted against the filling fraction of 4d sites. The arrow marks the jump of formation energies from HH TiFe_0.67_Cu_0.33_Sb with *x* + *y* = 1 to vacancy‐filling TiFe*_x_* Cu*_y_* Sb with *x* + *y* > 1.

The semiconducting behavior of, for example, HH TiCoSb can be explained by analyzing the electron configuration in its orbitals: The d‐orbital interaction of the two transition metals Ti and Co opens the bandgap in the HH structure.^[^ ^42^, ^43^, ^44^ ^]^ The experimental absence of the 17‐electron HH compound TiFeSb, which contains one electron less than the 18‐electron TiCoSb, suggests that a deficit in electron count could disrupt the orbitals necessary for stability. A straightforward remedy is doping TiFeSb with copper, which typically assumes a + 1 oxidation state. Copper is also chemically compatible with Fe, as they share similar electronegativity and ionic radii. Upon doping, the TiFeSb HH matrix transitions into a structure with partially filled vacancies, where some 4d sites become occupied (**Figure** **1**a). If the interaction between the 4c and 4d sites is strong enough, it may lead to significant crystal‐field splitting between the lower energy *t*_2g_ and higher energy *e*_g_ states. Additionally, positioning the Fermi level between the completely filled *t*_2g_ and empty *e*_g_ bands should help stabilize the doped crystal structure.

With these insights, we determine the stoichiometry for TiFeSb‐Cu alloys to achieve a stable structure with a bandgap. The composition for these materials is represented as TiFe*_x_* Cu*_y_* Sb. To accommodate the potential intermixing of Fe and Cu atoms, we denote the atomic positions as TiFe*_x_*_4c_Fe*_x_*_4d_Cu*_y_*_4c_Cu*_y_*_4d_Sb, where *x*_4c_ (or *y*_4c_) and *x*_4d_ (or *y*_4d_) specify the respective concentrations of Fe (or Cu) at the 4c and 4d lattice sites. These concentrations satisfy the equation *x*_4c_ + *x*_4d_ = *x* (or *y*_4c_ + *y*_4d_ = *y*). Structurally, it is energetically favorable for the 4c sites to be preferentially occupied, which helps to stabilize the HH framework and minimize Coulombic repulsion between the 4c and 4d sites. Electronically, the formation of a bandgap depends on properly populating the Fe *t*_2g_ sub‐orbitals when Fe is positioned at the 4d sites, leading to *y* = 2*x*_4c_ − 2*x*_4d_ −1 = 0 to ensure the correct electron count for the bandgap formation (see the Supporting Information Section 2 for more information).

**Table** **1** presents four types of stoichiometries classified by 4d occupation. Type‐I systems are the foundational scenarios in which the 4d sites remain vacant (*x*_4d_ = *y*_4d_ = 0), resulting in the HH TiFe_0.67_Cu_0.33_Sb with 18 electrons. Interestingly, the Fe concentration (*x* = 0.67) is less than the stoichiometric amount (*x* = 1.0) in the reference material TiFeSb. Type‐II materials are characterized by the partial occupation of 4d sites by Fe atoms (i.e., *x*_4d_ ≠ 0 but *y*_4d_ = 0). With each specified concentration of Fe (i.e., *x* value), the site occupations for Fe (*x*_4c_ and *x*_4d_) and Cu (*y*_4c_ and *y*_4d_) are accordingly determined. We list six example stoichiometries, spanning *x* values of 0.7, 0.8, 0.9, 1.0, 1.25, and 1.50. The endpoint TiFe_1.50_Cu_0.00_Sb reduces to ternary TiFe_1.50_Sb, a semiconductor proposed by Snyder.^[^ ^45^ ^]^ Type‐III materials are distinguished by the presence of Cu atoms at some 4d sites (i.e., *x*_4d_ = 0 but *y*_4d_ ≠ 0). A special stoichiometry, TiFe_1.00_Cu_1.00_Sb, is referred to as an FH system. Type‐IV accommodates both Fe and Cu in the 4d sites, offering a more realistic representation of atom distribution in practical materials. Here, the *y*_4d_ values are manually specified because they remain underdetermined based on the design rules.

**Table 1:** Illustration of the atomic stoichiometry of TiFe*_x_* Cu*_y_* Sb semiconductors. The formula of TiFe*_x_*_4c_Fe*_x_*_4d_Cu*_y_*_4c_Cu*_y_*_4d_Sb specifies the site occupation patterns.

Type	Site occupations					Examples					
	Assumption	Fe, *x*_4c_	Fe, *x*_4d_	Cu, *y*_4c_	Cu, *y*_4d_	*x*	Fe, *x*_4c_	Fe, *x*_4d_	Cu, *y*_4c_	Cu, *y*_4d_	Formula
I	*x*_4*d*_ = 0 *y*_4*d*_ = 0 (*x* = 0.667)	$\frac{2}{3}$	0	$\frac{1}{3}$	0	0.67	0.67	0.00	0.33	0.00	TiFe_0.67_Cu_0.33_Sb
II	$x_{4d}\neq 0$ *y*_4*d*_ = 0 (0.667 < *x* ≤ 1.5)	$\frac{2x+2}{5}$	$\frac{3x-2}{5}$	$\frac{3-2x}{5}$	0	0.70 0.80 0.90 1.00 1.25 1.50	0.68 0.72 0.76 0.80 0.90 1.00	0.02 0.08 0.14 0.20 0.35 0.50	0.32 0.28 0.24 0.20 0.10 0.00	0.00 0.00 0.00 0.00 0.00 0.00	TiFe_0.70_Cu_0.32_Sb TiFe_0.80_Cu_0.28_Sb TiFe_0.90_Cu_0.24_Sb TiFe_1.00_Cu_0.20_Sb TiFe_1.25_Cu_0.10_Sb TiFe_1.50_Cu_0.00_Sb
III	*x*_4*d*_ = 0 $y_{4d}\neq 0$ (0.667 < *x* ≤ 1.0)	*x*	0	1 − *x*	3*x* − 2	0.70 0.73 0.75 0.76 0.78 0.80 0.90 1.00	0.70 0.73 0.75 0.76 0.78 0.80 0.90 1.00	0.00 0.00 0.00 0.00 0.00 0.00 0.00 0.00	0.30 0.27 0.25 0.24 0.22 0.20 0.10 0.00	0.10 0.19 0.25 0.28 0.34 0.40 0.70 1.00	TiFe_0.70_Cu_0.40_Sb TiFe_0.73_Cu_0.46_Sb TiFe_0.75_Cu_0.50_Sb TiFe_0.76_Cu_0.52_Sb TiFe_0.78_Cu_0.56_Sb TiFe_0.80_Cu_0.60_Sb TiFe_0.90_Cu_0.80_Sb TiFe_1.00_Cu_1.00_Sb
IV	$x_{4d}\neq 0$ $y_{4d}\neq 0$ (0.667 < *x* ≤ 1.0)	$\frac{2x+2+y_{4d}}{5}$	$\frac{3x-2-y_{4d}}{5}$	$\frac{3-2x-y_{4d}}{5}$	*y*_4*d*_	0.70 0.80 0.80 0.90 0.90	0.69 0.73 0.74 0.79 0.80	0.01 0.07 0.06 0.11 0.10	0.31 0.27 0.26 0.21 0.20	0.05 0.05 0.10 0.15 0.20	TiFe_0.70_Cu_0.36_Sb TiFe_0.80_Cu_0.32_Sb TiFe_0.80_Cu_0.36_Sb TiFe_0.90_Cu_0.36_Sb TiFe_0.90_Cu_0.40_Sb

The systems are subsequently modeled using density‐functional theory to calculate their formation energies (Figure 1c). Atomic occupation disorder, which is inevitable due to the irregular Fe‐Cu mixing and stochastic partial filling of 4d sites, is accounted for using large supercells. The HH TiFe_0.67_Cu_0.33_Sb exhibits a negative formation energy of −135 meV per atom, indicating an energy gain when the compound is synthesized from its elemental constituents. Above the half‐filling threshold (i.e., *x* + *y* > 1), all structures exhibit higher formation energies, suggesting they are comparatively less stable than TiFe_0.67_Cu_0.33_Sb. For instance, within the Type‐II materials, TiFe_0.70_Cu_0.32_Sb displays a formation energy of −120 meV per atom, while TiFe_0.80_Cu_0.28_Sb shows −114 meV per atom.

The increased formation energies of vacancy‐filling systems can be ascribed to the Coulombic repulsion between the atoms at the 4c and 4d sites. Each atom at a 4d site experiences repulsion from six neighboring 4c atoms, profoundly impacting stability. This understanding allows for a simple organization of formation energies relative to the filling fraction of 4d sites, as shown in Figure 1d: Type‐II and Type‐IV compounds follow a lower‐energy pathway, while Type‐III compounds are charted along a higher energy trajectory with less stability, approximately four meV/atom higher. For example, when comparing compounds with equal Fe content, Type‐II TiFe_0.70_Cu_0.32_Sb exhibits the lowest and most favorable formation energy compared to Type‐III TiFe_0.70_Cu_0.40_Sb and Type‐IV TiFe_0.70_Cu_0.36_Sb. The more favorable stability of Type‐II systems is attributed to their smaller Cu concentrations (and consequently fewer 4c‐4d atomic pairs with Coulombic repulsion). Interestingly, Type‐II and Type‐I systems, which have favorable formation energies, comply with the Slater‐Pauling rule of six electrons per atom. For Type‐II systems, we also intentionally consider the disruption of Fe‐Cu occupation patterns, which aligns the formation energies with the less stable trajectory (as indicated by the dashed bars in Figure 1c).

### 2.2 Experimental Characterization of Crystal Structure

**Figure 2:**
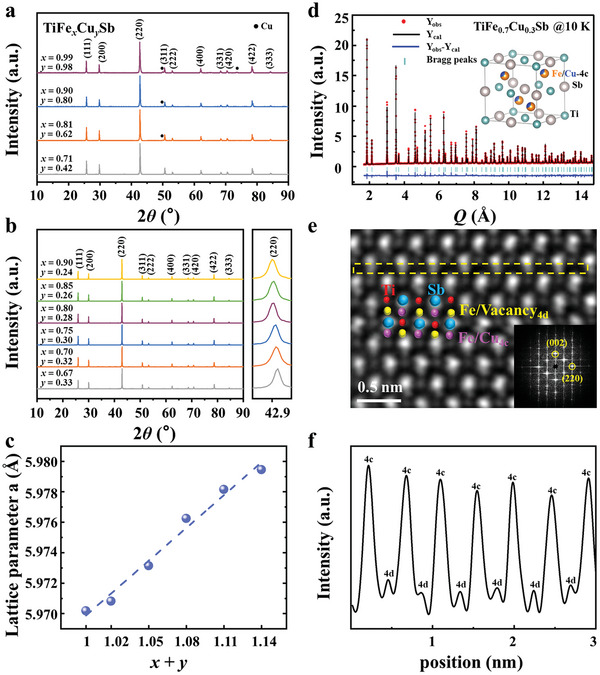
Structural properties of TiFe*_x_* Cu*_y_* Sb. a) XRD patterns of type‐III samples. b) XRD results of type‐I and type‐II systems. The right panel displays a magnified view of the (220) peak. c) Variation of lattice parameters concerning the vacancy filling levels. d) Neutron diffraction pattern of Type‐II TiFe_0.7_Cu_0.3_Sb at 300 K. e) High‐resolution HAADF image corrected for aberration, viewed along the [110] direction in Type‐II TiFe_0.80_Cu_0.28_Sb. The inset shows the SAED pattern along the [110] direction. f) Intensity profile extracted from the horizontal line in the HAADF image shown in subplot (e).

According to the theoretical predictions, we synthesized various samples and subsequently characterized their structural properties. Type‐III systems are prepared according to the theoretical stoichiometries, and X‐ray diffraction (XRD) finds that the annealed samples have a strong propensity to precipitate copper, as evidenced by specific peaks in **Figure** **2**a. In contrast, the XRD patterns for Type‐I and Type‐II systems show a clean crystalline structure that aligns with a face‐centered cubic, MgAgAs‐type lattice (Figure 2b). The real sample compositions of Type‐I and Type‐II systems are very close to their nominal compositions, agreeing well with our chemical composition analysis by the electron probe micro‐analysis (EPMA, see Table S1, Supporting Information). Our subsequent discussions will focus on the stable Type‐I and II systems, specifically examining two examples with Fe concentration levels of *x* = 0.7 and 0.8.

The crystalline quality of our samples is characterized using various experimental techniques. Figure 2b illustrates that the (220) diffraction peak shifts monotonically to lower angles as vacancy‐filling levels increase, indicating a gradual lattice expansion. This observation aligns well with the measured lattice constants shown in Figure 2c. Neutron diffraction refinement of TiFe_0.7_Cu_0.3_Sb, depicted in Figure 2d, further reveals that the sample consists of a single pure phase. Notably, the neutron‐determined Cu concentration of 0.3 is slightly lower than the theoretical value of 0.32; however, this discrepancy does not significantly affect the crystalline quality. This implies that these vacancy‐filling Heusler alloys can tolerate stoichiometric deviations from the critical values typically necessary for semiconductors. Neutron measurements also reveal that the 4c (4d) sites are fully occupied (empty) in TiFe_0.7_Cu_0.3_Sb, imparting characteristics similar to an HH, except for the random mixing of Fe and Cu at the 4c sites. The crystal structure and atomic occupancy of TiFe_0.80_Cu_0.28_Sb, which exhibits the highest thermoelectric performance among all samples, are further analyzed using transmission electron microscopy. A typical dark‐field image and its associated energy‐dispersive spectrum (Figure S1, Supporting Information) reveal a very homogeneous distribution of Ti, Fe, Cu, and Sb elements, with no clustering within the sample.

Given that the Fe and Cu concentrations in TiFe_0.80_Cu_0.28_Sb exceed that of HH, we further investigate the occupation patterns of the 4c and 4d sites in this sample. Figure 2e presents an image of atom‐resolved high‐angle annular dark‐field scanning transmission electron microscopy (HAADF‐STEM), it can be seen both 4c and 4d positions can be occupied, and the selected area electron diffraction (SAED) pattern along the [110] direction confirms a face‐centered cubic structure. The atomic intensity profile shown in Figure 2f reveals a notable disparity between high‐intensity and low‐intensity peaks, indicating a higher propensity for filling 4c sites than 4d sites, the sample should be HH‐like structures ($F\bar{4}3m$). The X‐ray diffraction (XRD) patterns are refined using the Rietveld method,^[^ ^46^, ^47^ ^]^ applying various structural models to best represent the crystal structure (Figure S2, Supporting Information). Our findings confirm that TiFe_0.80_Cu_0.28_Sb is a single‐phase material with $F\bar{4}3m$ space group. Both 4c and 4d sites exhibit mixed occupation by Fe and Cu. However, Cu preferentially occupies the 4c sites in particular samples, agreeing with the theoretical predictions.

### 2.3 Influence of Vacancy‐Filling Levels on Material Properties

Theoretical simulations suggest that metallicity can emerge in TiFe*_x_* Cu*_y_* Sb materials at high vacancy‐filling levels. As depicted in Figure 1b, TiFe_0.67_Cu_0.33_Sb functions effectively as a semiconductor, exhibiting a bandgap of 0.56 eV, although this may be subject to underestimation. As we transition to Type‐II systems with increased atomic concentrations, electronic states begin to appear within the intrinsic bandgap region, suggesting a potential shift from a good semiconductor to partial metallicity. For instance, TiFe_1.00_Cu_0.20_Sb sees its bandgap disappear, displaying characteristics akin to those of degenerate semiconductors. Moreover, deviations from the established Fe‐Cu site occupation pattern, inevitable in practical experiments, significantly enhance these metallic characteristics.

**Figure 3:**
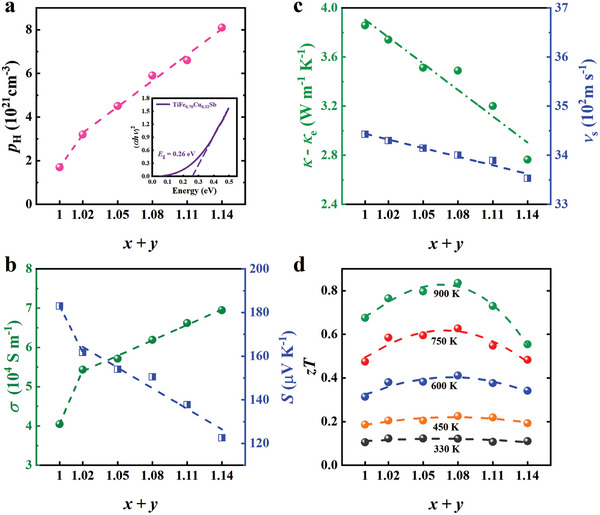
Electrical transport properties. a) Hall carrier concentration, *p*_H_. The inset shows the absorption spectrum of TiFe_0.70_Cu_0.32_Sb and the determination of the bandgap. b) Electrical conductivity, *σ*, and Seebeck coefficient, *S*. c) Lattice thermal conductivity, *κ*_L_ = *κ – κ*_e_, and the averaged sound velocity, versus d) thermoelectric figure of merit, z*T*. Note the “jump” features for *p*_H_, *σ*, and *S*.

The predicted variation in carrier concentrations is experimentally explored through Hall effect measurements at room temperature, as illustrated in **Figure** **3**a and Table S2 (Supporting Information). The carrier concentration, *p*_H_, increases from 1.7 × 10^21^ cm^−3^ in TiFe_0.67_Cu_0.33_Sb to 8.1 × 10^21^ cm^−3^ in TiFe_0.90_Cu_0.24_Sb. The electrical characteristics of all samples reveal the presence of p‐type charge carriers, aligning with our theoretical predictions (Figure 1b). Notably, the Hall results capture a distinct “jump” in behavior transitioning from the HH TiFe_0.67_Cu_0.33_Sb, with empty 4d sites, to the vacancy‐filling systems beginning with TiFe_0.70_Cu_0.32_Sb. As the vacancy filling levels increase, electrical conductivity rises, and the Seebeck coefficient falls (Figure 3b). Based on the measured Seebeck coefficient, the bandgap (*E*_g_) can be calculated using the formula *E*_g_ = 2*eS*_max_ *T*_max_,^[^ ^48^ ^]^ where *S*_max_ is the maximum Seebeck coefficient, *e* is the elementary charge, and *T*_max_ is the temperature at which *S*_max_ is observed. For instance, the calculated bandgap for TiFe_0.70_Cu_0.32_Sb is 0.30 eV, closely aligning with the optical bandgap of 0.26 eV derived from the absorption spectrum (inset of Figure 3a). The bandgap of all samples (calculated using the Goldsmid‐Sharp formula) decreases as the vacancy filling levels increase. This is consistent with the theoretical predictions, see Supporting Information Table S3 (Supporting Information).

Due to the Fe/Cu‐mixed occupation on 4c sites and the partially random filling of 4d sites, significant phonon scattering is expected in TiFe*_x_* Cu*_y_* Sb materials. The lattice thermal conductivity, determined by subtracting the electronic contribution from the total thermal conductivity, indeed shows a linear decrease with respect to the atomic concentrations, as illustrated in Figure 3c and the methods section. Figure 3c (also refer to Figure S3 and Table S4, Supporting Information) demonstrates that the average sound velocities decrease with the atomic concentrations.

As a function of the vacancy‐filling levels, we have observed an increase in electrical conductivity accompanied by a simultaneous reduction in both the Seebeck coefficient and thermal conductivity. These changes result in a significant compromise and synergy that profoundly impact the thermoelectric properties, as shown in Figure 3d. The highest figure of merit is achieved with the intermediate stoichiometry TiFe_0.80_Cu_0.28_Sb. Furthermore, these materials exhibit non‐trivial temperature‐dependent performances, which will be discussed in the following section.

### 2.4 Temperature‐Dependent Thermoelectric Properties

**Figure 4:**
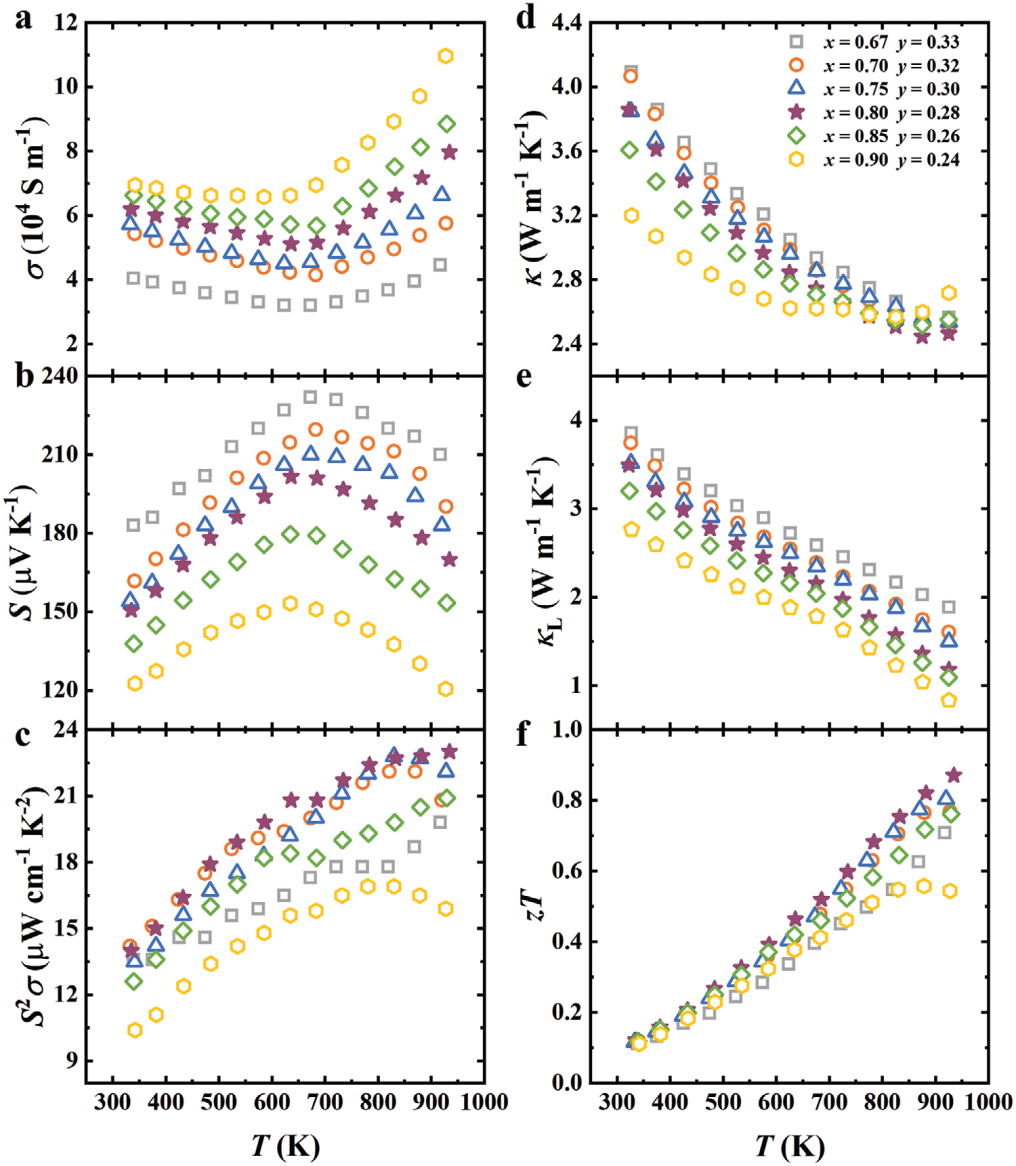
Temperature‐dependent thermoelectric properties. a) Electrical conductivity. b) Seebeck coefficient (*S*). c) Power factor. d) Total thermal conductivity, *κ*. e) Lattice thermal conductivity, *κ*_L_ = *κ – κ*_e_. f) Figure of merit, *zT*.

For all samples, the electrical conductivity as a function of temperature (**Figure** **4**a) shows a characteristic turning point between 600 and 700 K, a feature typical of heavily doped semiconductors. Before this turning point, the electrical conductivity decreases with increasing temperature due to enhanced phonon scattering, which impedes the mobility of the near‐free carriers (holes in these p‐type TiFe*_x_* Cu*_y_* Sb materials). Beyond the turning temperatures, however, the electrical conductivity begins to increase. This change is attributed to intrinsic excitations across the semiconductor's bandgap becoming significant at higher temperatures, which increases the overall carrier density and thus electrical conductivity. In contrast, the Seebeck coefficient (Figure 4b) exhibits an inverse temperature dependence compared to the electrical conductivity. The Seebeck coefficients of all samples exceed 120 µV K^−1^, with the standout TiFe_0.67_Cu_0.33_Sb material peaking at 232 µV K^−1^ at 673 K. Despite these high values, the bipolar effect at elevated temperatures serves as a significant limiting factor for the Seebeck coefficients.

The relationship between electrical conductivity and the Seebeck coefficient is further explored through the power factor, as presented in Figure 4c. Except for TiFe_0.90_Cu_0.24_Sb, which exhibits the highest degree of metallicity, the power factors of the other samples increase with temperature. This trend is particularly notable in systems with intermediate atomic concentrations, specifically Fe concentrations ranging from *x* = 0.7 to 0.8. For instance, the sample TiFe_0.80_Cu_0.28_Sb achieves an impressive thermoelectric power factor of 23 µW cm^−1^ K^−2^ at 923 K.

The total thermal conductivity (Figure 4d) decreases rapidly with rising temperatures up to 600–700 K, coinciding with the temperatures of intrinsic excitation. Beyond this range, the rate of decrease becomes less pronounced, particularly in the case of TiFe_0.90_Cu_0.24_Sb. This turning point is attributed to a significant increase in electronic thermal conductivity above the intrinsic excitation temperature (Figure S4, Supporting Information). Meanwhile, the lattice contribution to thermal conductivity, *κ*_L_, shows a monotonous decline (Figure 4e). Notably, *κ*_L_ values for all samples remain below 3.90 W m^−1^ K^−1^, significantly lower than that observed in conventional 18‐electron HH alloys without the partially filled vacancies.^[^ ^49^ ^]^

**Figure 5:**
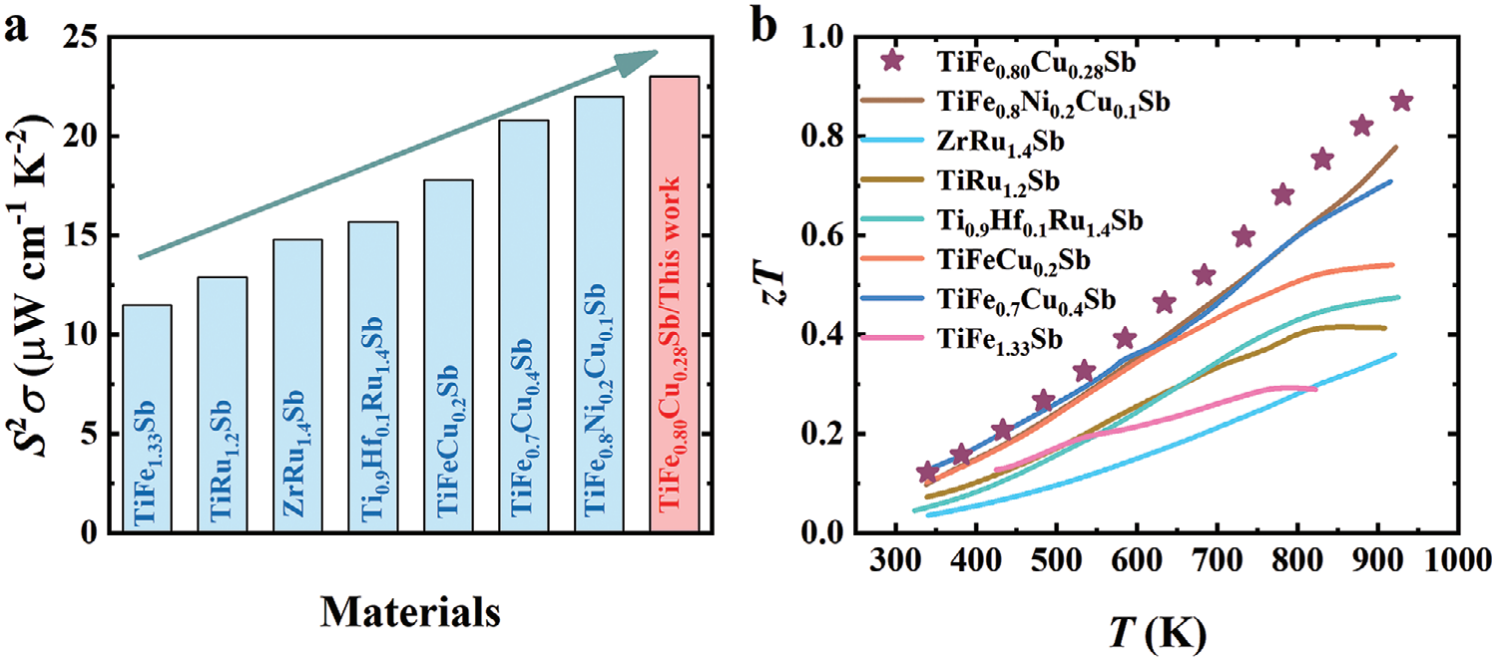
Comparative analysis on TiFe_0.80_Cu_0.28_Sb and other vacancy‐filling Heusler alloys.^[^ ^34^, ^36^, ^37^, ^38^, ^39^, ^40^, ^41^ ^]^ a) Maximum power factors. b) *zT* values.

Figure 4f compiles the *zT* values for all samples, highlighting that TiFe_0.80_Cu_0.28_Sb (with a peak *zT* = 0.87 at 923 K) outperforms others across the temperature spectrum investigated. This exceptional *zT* value is primarily attributed to its high power factor, closely linked to the carrier density. Notably, TiFe_0.80_Cu_0.28_Sb not only achieves the highest *zT* value among all the existing vacancy‐filling Heuslers but also exhibits the highest power factor (**Figure** **5**).

Good thermoelectric materials are typically heavily doped narrow‐gap semiconductors, traditionally achieved by doping with heterovalent elements. This study explores an alternative approach with quaternary Heuslers TiFe*_x_* Cu*_y_* Sb, which involves manipulating carriers by partially filling the vacant 4d sites. This vacancy‐filling method offers several advantages. First, it allows for continuous adjustments in stoichiometries, thereby continuously affecting carrier densities and electrical conductivities, significantly enriching the Heusler family. Second, the emergence of metallic behaviors due to non‐ideal site occupation in practical experiments suggests that this carrier doping should be considered as an intrinsic effect and effective approach. This method is distinctly different from the conventional approach of introducing heterovalent dopants. Third, since this doping strategy does not introduce additional complications, it can be integrated with other techniques to further optimize thermoelectric properties. For example, isoelectronic doping at the 4a and 4b sites may further reduce lattice thermal conductivity. Despite these advantages, the bipolar effect (not unique to TiFe*_x_* Cu*_y_* Sb) and low carrier mobility impose limitations on the thermoelectric performance.^[^ ^50^ ^]^

## 3 Conclusion

In summary, we have theoretically designed and experimentally synthesized a series of quaternary TiFe*_x_* Cu*_y_* Sb Heuslers. Structural characterizations reveal that the 4c sites preferentially host a random mix of Fe and Cu atoms, while the 4d sites are partially and randomly filled with the remaining atoms. The electrical conductivities of these materials transition from a relatively good semiconductor with low carrier concentration to a heavily degenerate semiconductor. Notably, an intermediate stoichiometry, TiFe_0.80_Cu_0.28_Sb, achieves a *zT* value of 0.87 at 923 K among the TiFe*_x_* Cu*_y_* Sb samples and also sets a record for all known vacancy‐filling Heuslers. This finding validates our objective to develop optimal thermoelectric materials by effectively bridging the gap from good to degenerate semiconductors and thus highlights the potential of vacancy‐filling Heuslers as promising high‐performance thermoelectric materials.

## 4 Experimental Section

### Theoretical Calculations

The density‐functional theory simulations were conducted utilizing the FHI‐aims code,^[^ ^51^ ^]^ employing the Strongly Constrained and Appropriately Normed (SCAN) functional.^[^ ^52^ ^]^ Complete relaxation was ensured of all crystal structures. Formation energy is calculated as the energy discrepancy between the compound and its constituent elemental phases, defined as *E* ^f^ = *E*(TiFe*_x_* Cu*_y_* Sb) – *E*(Ti) – *xE*(Fe) – *yE*(Cu) – *E*(Sb). To accurately represent atomic disorder, the simulations employed supercell models comprising over 300 atoms.

### Sample Synthesis

Polycrystalline samples with the nominal composition TiFe*_x_* Cu*_y_* Sb were synthesized through a combined process of high‐energy ball milling followed by spark plasma sintering (SPS). High‐purity raw materials (Ti pieces, 99.6%; Fe pieces, 99.9%; Cu wires, 99.9%; Sb rods, 99.999%) were weighed according to the nominal composition and loaded into a stainless‐steel tank under an argon atmosphere to prevent oxidation. The mixture was ball milled for 20 h using a SPEX 8000 m mixer per mill. Subsequently, the milled powders were transferred to a graphite die with an inner diameter of 10 mm. The samples were sintered by an SPS system (LABOX‐325GH‐C1, SINTERLAND, Japan). The sintering temperature was ramped up to 973 K in 30 min under an applied pressure of 50 MPa, and then held at this temperature for 10 min to consolidate the samples. The resulting sintered samples exhibited a relative mass density greater than 95% (see Table S5, Supporting Information). To improve the crystallinity and relieve internal stresses, the samples were subsequently annealed at 973 K for a duration of 48 h.

### Sample Characterization

Phase identification and crystal structure analysis were carried out with high‐resolution powder XRD patterns collected by a Rigaku SmartLab‐II diffractometer with Cu‐K_α_ radiation. EXPGUI software was used to refine the XRD pattern and analyze the atomic occupancies. The microstructures of the samples were examined by a TEM (JEM‐F200, JEOL, Japan) and a probe Cs‐corrected TEM (Themis ETEM, Thermo Fisher Scientific, USA). TEM specimens were prepared by mechanical slicing, polishing, and dimpling, followed by ion milling. Dark field TEM images and HAADF images were obtained using the STEM model. Energy‐dispersive X‐ray spectroscopy was employed to determine the distribution of elements at the nanoscale. The chemical compositions of the samples were measured by an EPMA (JXA‐IHP200F, Japan). Neutron powder diffraction patterns of TiFe_0.7_Cu_0.3_Sb were obtained by using the Super High‐Resolution Powder Diffractometer, BL08 SuperHRPD, at the Material and Life Science Experimental Facility (MLF) of Japan Proton Accelerator Research Complex (J‐PARC). Approximately 3 grams of samples were ground into powder in agate mortar and loaded into a vanadium can for NPD measurements. The NPD patterns were analyzed using the Z‐Rietveld software.^[^ ^53^ ^]^

### Transport Properties Measurements

Seebeck coefficients and resistivities were simultaneously measured by a ULVAC ZEM‐3 system through the four‐probe method. The total thermal conductivity *κ* was calculated by *κ* = *λρC*_P_, where *λ* is the thermal diffusivity, *ρ* is the mass density of the sample, and *C*_P_ is the specific heat capacity. The thermal diffusivity *λ* of a disk‐shaped sample, 10 mm in diameter and ≈1 mm in thickness was measured using a laser pulse method by an LFA 457 apparatus (NETZSCH, Germany). The room‐temperature Hall coefficient *R*_H_ was measured by a physical property measurement system (PPMS, Quantum Design, USA), and the Hall carrier concentration *p*_H_ was calculated according to *p*_H_ = 1/(*eR*_H_) where e is the elementary charge. The room‐temperature sound velocity was measured by the ultrasonic material characterization system (UMS‐100, TECLAB, France). The electronic thermal conductivity *κ*_e_ is determined using the Wiedemann‐Franz law, *κ*_e_ = *LσT*, where *L* is the Lorenz number calculated by *L* = (1.5 + $e^{-\frac{|S|}{116}}$)×10^−8^ W Ω K^−2^.^[^ ^54^ ^]^ The lattice thermal conductivity *κ*‐*κ*_e_ is subsequently deduced by subtracting *κ*_e_ from the total thermal conductivity.

### Bandgap Measurements

The absorbance coefficient (*α*) was measured using the Fourier transform infrared spectrometry (FTIR, Nicolet iS50, USA), and the bandgap (*E*_g_) was then estimated by the Tauc plot method, (*αhν*)^1/n^ = B(*hν* – *E*), where *h* is the Planck constant, *ν* is the photon's frequency, *hν* is the photon energy, and B is the proportionality constant. The value of the exponent n depends on the nature of the electron transition type (*n* = 0.5 for the direct bandgap and *n* = 2 for the indirect bandgap).

## Conflict of Interest

The authors declare no conflict of interest.

## Author Contributions

W.‐M.H. and S.Y. equally contributed to this work. J.L. performed conceptualization, supervision, funding acquisition, writing, reviewing, and editing. W.‐M.H. prepared the samples and wrote the original draft. S.Y. helped prepare the samples. Y.‐B.Z. carried out the theoretical calculations, writing, reviewing, and editing. Z.‐R.D. measured the electrical properties, writing, reviewing, and editing. B.‐R.Z., M.H., S.T., and J.M. measured and analyzed neutron diffraction data. Q.‐Z.L. and B.‐H.G. performed the microstructure analysis on TEM. J.‐Y.Z. was involved in data curation.

## Supporting information

Supporting Information

## Data Availability

The data that support the findings of this study are available from the corresponding author upon reasonable request.
