# Supplementary material for: Strategic Design and Mechanistic Understanding of Vacancy‐Filling Heusler Thermoelectric Semiconductors
Source: Adv Sci (Weinh). 2024 Sep 3;11(40):2407578. doi: 10.1002/advs.202407578 (PMC11516113; doi:10.1002/advs.202407578)
Supplement: Supplementary file 1 — Supporting Information [file ADVS-11-2407578-s001.docx]

Supporting Information

Strategic Design and Mechanistic Understanding of Vacancy-Filling Heusler Thermoelectric Semiconductors

Weimin Hu, Song Ye, Qizhu Li, Binru Zhao, Masato Hagihala, Zirui Dong,* Yubo Zhang,* Jiye Zhang, Shuki Torri, Jie Ma, Binghui Ge, Jun Luo*

Weimin Hu, Song Ye, Zirui Dong, Jiye Zhang

School of Materials Science and Engineering, Shanghai University, Shanghai 200444, China
E-mail: zirui@shu.edu.cn

Qizhu Li, Binghui Ge

Institutes of Physical Science and Information Technology, Anhui University, 111 Jiulong Road, Hefei, 230601, China

Binru Zhao, Jie Ma

Key Laboratory of Artificial Structures and Quantum Control, School of Physics and Astronomy, Shanghai Jiao Tong University, Shanghai 200240, China

Masato Hagihala, Shuki Torri

Institute of Materials Structure Science, High Energy Accelerator Research Organization (KEK), Tokai, Ibaraki, 319-1106, Japan

Yubo Zhang

Minjiang Collaborative Center for Theoretical Physics, College of Physics and Electronic Information Engineering, Minjiang University, Fuzhou, China

E-mail: yubo.drzhang@mju.edu.cn

Jun Luo

Interdisciplinary Materials Research Center, School of Materials Science and Engineering, Tongji University, Shanghai 201804, China

E-mail: junluo@tongji.edu.cn

**Section 1**

**Room-temperature elastic properties**

Average sound velocity *v*_s_ can be extracted from

$$\begin{aligned} v_{s}=\left[ \frac{1}{3}\left( \frac{1}{v_{l}^{3}}+\frac{2}{v_{t}^{3}} \right) \right]^{-\frac{1}{3}}\#(S1) \end{aligned}$$

Debye temperature *θ*_D_

$$\begin{aligned} \theta_{D}=\frac{h}{k_{B}}\left( \frac{3N}{4\pi V} \right)^{\frac{1}{3}}v_{s}\#(S2) \end{aligned}$$

bulk modulus *B*

$$\begin{aligned} B=\frac{\rho\left( 3v_{l}^{2}-4v_{t}^{2} \right)}{3}\#(S3) \end{aligned}$$

Grüneisen parameter *g*_G_

$$\begin{aligned} \gamma_{G}=\frac{3}{2}\left( \frac{3v_{l}^{2}-4v_{t}^{2}}{v_{l}^{2}+2v_{s}^{2}} \right)\#(S4) \end{aligned}$$

where *V* is the unit-cell volume, *N* is the number of atoms in a unit cell, *h* represents the Planck constant, and *ρ* is the mass density. *v*_l_ and *v*_t_ are the longitudinal and transverse sound velocities, respectively.

**Section 2**

The fundamental principle of our design strategy is to achieve full occupation of orbitals or sub-orbitals. To accommodate the possible Fe and Cu occupation variations, we define atomic occupations as $\mathrm{Fe}_{x_{4c}}\mathrm{Fe}_{x_{4d}}\mathrm{Cu}_{y_{4c}}\mathrm{Cu}_{y_{4d}}\mathrm{TiSb}$, where $x_{4c}+x_{4d}=x$, and $y_{4c}+y_{4d}=y$ represent the Fe and Cu concentrations at the 4c and 4d sites, respectively. Now, let's assign the electron occupations for the spin-up and spin-down channels, taking into account the following considerations:

- $\mathrm{Fe}_{x_{4c}}$: We allocate 5 electrons to the spin-up channel (5$x_{4c}$) to fully occupy all five d orbitals, considering the spin degree of freedom, with the remaining 3 electrons assigned to the spin-down channel (3$x_{4c}$).
- $\mathrm{Fe}_{x_{4d}}$: Since the d-orbitals at 4d sites have six neighbors—more than those at 4c sites—the occupation is typically lower, as supported by DFT simulations. Here, we ensure full occupation of the t_2g_ sub-orbitals with 3 electrons, assigning them to the spin-up channel (3$x_{4d}$) and the remaining electrons to the spin-down channel (5$x_{4d}$).
- TiSb: The two atoms contribute a total of 9 electrons, we prioritize full occupation of the s and p orbitals in the spin-up channel with 4 electrons (1 for the s orbital and 3 for the p orbitals), directing the leftover 5 electrons to the spin-down channel.
- $\mathrm{Cu}_{y_{4c}}\mathrm{Cu}_{y_{4d}}$: Because the spin up orbitals and sub-orbitals (s, p, d, and t_2g_) have been fully filled through the above steps, all of Cu's electrons are assigned to the spin-down channel, summed as $y_{4c}+y_{4d}=y$.

Spin compensation between the two channels is essential for inducing a band gap, and we equate the total electron occupations in both spin channels.

- Spin up: 5$x_{4c}$ + 3$x_{4d}$ + 4
- Spin down: 3$x_{4c}$ + 5$x_{4d}$ + 5 + $y$
- Spin compensation: 5$x_{4c}$ + 3$x_{4d}$ + 4 = 3$x_{4c}$ + 5$x_{4d}$ + 5 + $y$

Finally, we derive the relationship $y= 2x_{4c}-2x_{4d}-1$. It should be noted that there may be some ambiguity in the electron assignment during the design procedure. However, our design rule effectively encapsulates the critical gapping mechanism, particularly emphasizing the electron counting rules characteristic of vacancy-filling Heusler alloys.

**Figures and tables**

**
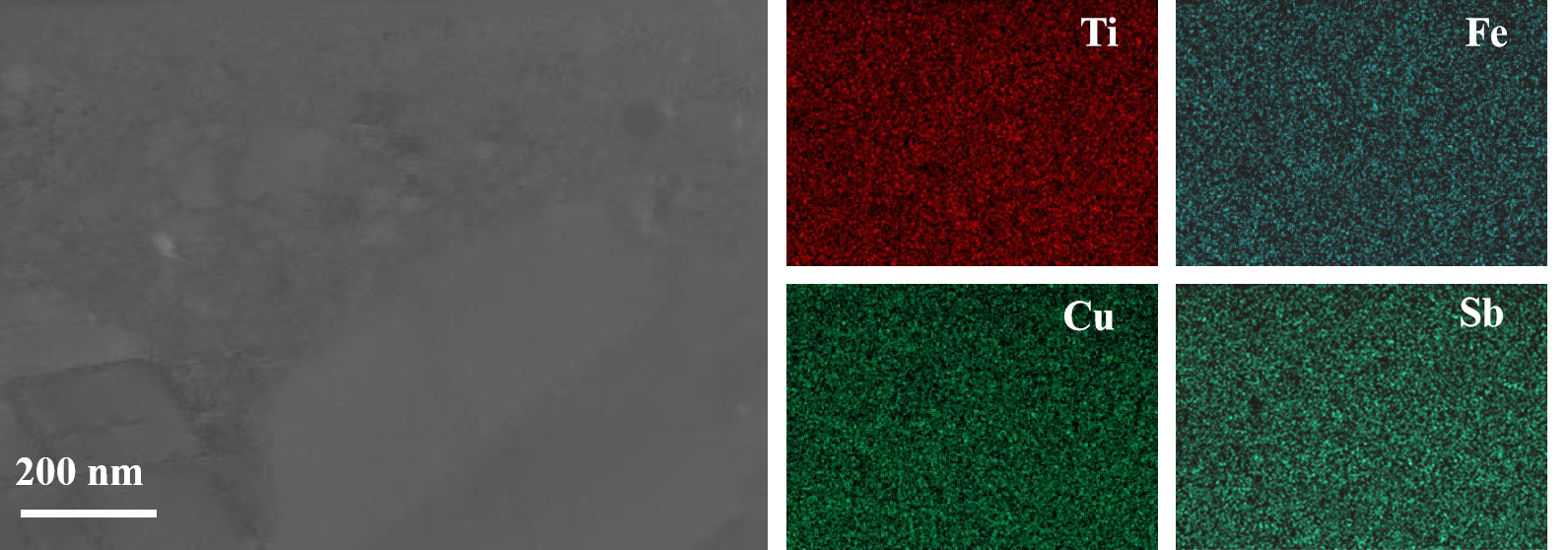
**

**Figure S1.** Dark field STEM image and corresponding EDS mappings of the TiFe_0.80_Cu_0.28_Sb sample.





**Figure S2.** XRD pattern of the TiFe_0.80_Cu_0.28_Sb sample together with the results of the crystal structure refinement.





**Figure S3.** Average (*v*_s_), longitudinal (*v*_l_), and transverse (*v*_t_) sound velocities of the TiFe*_x_*Cu*_y_*Sb samples.





**Figure S4.** Electronic thermal conductivities of the TiFe*_x_*Cu*_y_*Sb samples.

**Table S1.** Measured compositions of TiFe*_x_*Cu*_y_*Sb samples by EPMA.

| Nominal composition | Measured composition | | | | |
| --- | --- | --- | --- | --- | --- |
|  | Ti (at. %) | Fe (at. %) | Cu (at. %) | Sb (at. %) |  |
| TiFe0.67Cu0.33Sb | 33.254 | 22.656 | 10.719 | 33.371 | TiFe0.681Cu0.322Sb1.003 |
| TiFe0.70Cu0.32Sb | 33.189 | 23.232 | 10.365 | 33.214 | TiFe0.699Cu0.312Sb1.000 |
| TiFe0.75Cu0.30Sb | 32.313 | 23.956 | 10.285 | 33.446 | TiFe0.741Cu0.318Sb1.035 |
| TiFe0.80Cu0.28Sb | 32.204 | 26.212 | 8.875 | 32.708 | TiFe0.814Cu0.276Sb1.015 |
| TiFe0.85Cu0.26Sb | 32.096 | 27.469 | 8.179 | 32.256 | TiFe0.856Cu0.255Sb1.005 |
| TiFe0.90Cu0.24Sb | 31.587 | 28.887 | 7.406 | 32.118 | TiFe0.915Cu0.234Sb1.017 |

**Table S2.** Carrier concentrations (*p*_H_) of the TiFe*_x_*Cu*_y_*Sb samples at 300 K.

| Sample | *p*_H_ (×10^21^ cm^-3^) |
| --- | --- |
| TiFe_0.67_Cu_0.33_Sb | 1.7 |
| TiFe_0.70_Cu_0.32_Sb | 3.2 |
| TiFe_0.75_Cu_0.30_Sb | 4.5 |
| TiFe_0.80_Cu_0.28_Sb | 5.9 |
| TiFe_0.85_Cu_0.26_Sb | 6.6 |
| TiFe_0.90_Cu_0.24_Sb | 8.1 |

**Table S3.** Band gaps of TiFe*_x_*Cu*_y_*Sb samples.

| Sample | *E*g (eV) |
| --- | --- |
| TiFe_0.67_Cu_0.33_Sb | 0.31 |
| TiFe_0.70_Cu_0.32_Sb | 0.30 |
| TiFe_0.75_Cu_0.30_Sb | 0.28 |
| TiFe_0.80_Cu_0.28_Sb | 0.26 |
| TiFe_0.85_Cu_0.26_Sb | 0.23 |
| TiFe_0.90_Cu_0.24_Sb | 0.19 |

**Table S4.** Measured longitudinal (*v*_l_), transverse (*v*_t_) and average (*v*_s_) sound velocities, and estimated Debye temperature (*θ*_D_), bulk modulus (*B*) and Grüneisen parameter (*γ*_G_) for TiFe*_x_*Cu*_y_*Sb samples at room temperature.

| Sample | *v*_l_ (m s^-1^) | *v*_t_ (m s^-1^) | *v*_s_ (m s^-1^) | *θ*_D_ (K) | *B* (GPa) | *γ*_G_ |
| --- | --- | --- | --- | --- | --- | --- |
| TiFe_0.67_Cu_0.33_Sb | 5762 | 3082 | 3443 | 272.6 | 144.6 | 1.62 |
| TiFe_0.70_Cu_0.32_Sb | 5746 | 3070 | 3429 | 272.4 | 144.8 | 1.63 |
| TiFe_0.75_Cu_0.30_Sb | 5713 | 3056 | 3413 | 271.1 | 143.7 | 1.62 |
| TiFe_0.80_Cu_0.28_Sb | 5706 | 3043 | 3400 | 269.9 | 145.3 | 1.63 |
| TiFe_0.85_Cu_0.26_Sb | 5659 | 3035 | 3389 | 269.1 | 142.9 | 1.62 |
| TiFe_0.90_Cu_0.24_Sb | 5600 | 3002 | 3352 | 266.1 | 141.0 | 1.62 |

**Table S5.** Measured and calculated mass densities of the TiFe*_x_*Cu*_y_*Sb samples.

| Sample | Measured density  (g·cm^-1^) | Theoretical density  (g·cm^-1^) | Relative density  (%) |
| --- | --- | --- | --- |
| TiFe_0.67_Cu_0.33_Sb | 7.043 | 7.120 | 98.92 |
| TiFe_0.70_Cu_0.32_Sb | 7.082 | 7.150 | 99.05 |
| TiFe_0.75_Cu_0.30_Sb | 7.120 | 7.189 | 99.04 |
| TiFe_0.80_Cu_0.28_Sb | 7.190 | 7.225 | 99.51 |
| TiFe_0.85_Cu_0.26_Sb | 7.240 | 7.266 | 99.64 |
| TiFe_0.90_Cu_0.24_Sb | 7.287 | 7.308 | 99.71 |
